# GIST of the stomach masquerading as recurrent falls in an older adult: a case report and review

**DOI:** 10.1186/s12876-021-01964-6

**Published:** 2021-10-18

**Authors:** Louis Y. Tee, Lynette Sim, Li Feng Tan, Jeffrey Lum, Santhosh Kumar Seetharaman

**Affiliations:** 1 Division of Healthy Ageing, Alexandra Hospital, National University Health System, 378 Alexandra Road, Singapore, 159964 Singapore; 2 Department of Pathology, National University Hospital, National University Health System, Singapore, Singapore

**Keywords:** GIST, Older adult, Atypical presentation, Falls, Case report

## Abstract

**Background:**

Gastric tumors become increasingly prevalent with advanced age but can be challenging to diagnose in older adults who may present with non-specific symptoms. Here, we report a rare case of an occult gastric tumor associated with mesenteric panniculitis that presented with recurrent falls precipitated by vertigo.

**Case presentation:**

We describe a diagnostically challenging case of cryptogenic gastric tumor associated with mesenteric panniculitis in a 74-year-old female who presented with abdominal bloating and recurrent falls precipitated by vertigo, dehydration, acute kidney injury and electrolyte deficiencies, but had no alarm symptoms. Her symptoms resolved after laparoscopic wedge resection of the gastric tumor.

**Conclusions:**

Our case highlights that while alarm symptoms such as dysphagia, weight loss, gastrointestinal bleeding and vomiting are considered indications for endoscopy, clinicians should also maintain a high index of suspicion for gastric tumors in older patients who may present with atypical symptoms.

## Background

While current guidelines suggest selecting patients for endoscopy based on alarm symptoms such as dysphagia, weight loss, gastrointestinal bleeding, and vomiting [1–4], approximately 25% of patients with upper gastrointestinal tumors have no alarm symptoms [5]. Furthermore, even though older adults have an increased risk of gastric tumors due to advanced age and increased prevalence of *Helicobacter pylori* infections, they may present with mild or atypical symptoms, resulting in delayed diagnoses of upper gastrointestinal malignancies [6]. We describe a diagnostically challenging case of cryptogenic gastric tumor associated with mesenteric panniculitis in an older adult who presented with recurrent falls precipitated by vertigo but had no alarm symptoms.

## Case presentation

A 74-year-old Malay woman presented to the emergency department for recurrent falls precipitated by intermittent vertiginous giddiness, resulting in three hospital admissions in the past six months. She reported that the falls occurred at home when she was performing routine chores such as hanging clothes to dry while doing laundry and were precipitated by sudden intermittent attacks of vertigo of varying durations, ranging from a few minutes to one hour. The sudden bouts of vertigo caused to her lose balance and land on her buttocks. During her previous admissions to hospital, her giddiness was attributed to possible chronic vestibulopathy, but her symptoms recurred despite taking regular oral betahistine hydrochloride 24 mg twice a day and cinnarizine 25 mg three times a day. In fact, she was just discharged from hospital three days prior. While she reported intermittent abdominal bloating after meals for the past six months, she denied any weight loss, melena, hematochezia, dysphagia or vomiting. She denied any loss of consciousness, limb weakness, numbness or injuries, and she had no hearing loss, tinnitus, ear discharge or trauma to her ears.

Her past medical history includes hypertension, dyslipidemia, and poorly controlled type 2 diabetes mellitus, with a glycosylated hemoglobin (HbA1c) level of 8.9%, and mild cognitive impairment. Her regular oral medications included metformin 500 mg twice a day, glipizide 5 mg twice a day, lisinopril 20 mg once a day, and simvastatin 10 mg once a day. She had no personal or family history of cancer and had no previous surgeries. She never smoked, was teetotal, and did not consume supplements or traditional medicines.

On examination, she was afebrile and hemodynamically stable but lethargic; her tongue was dry, and her skin turgor was poor. Orthostatic hypotension was observed. Her abdomen was grossly distended with mild generalized tenderness, but was soft, with no guarding, rebound tenderness, palpable masses, succession splash, or ascites. Digital rectal examination was unremarkable, with no hematochezia, melena, masses or impacted stool. A thorough neurological examination, including vestibular tests, was performed to evaluate for a neurological cause for the vertigo. The neurological exam and gait were normal: There was no limb weakness, nystagmus, dysmetria, dysdiadochokinesia, rigidity, tremors or bradykinesia. Dix-Hallpike and head thrust tests were negative. Cardiac examination revealed no cardiac murmur or carotid bruits. Her vision and hearing were intact, and she had no knee osteoarthritis or injuries. Otoscopy was normal, revealing intact tympanic membranes.

**Fig. 1:**
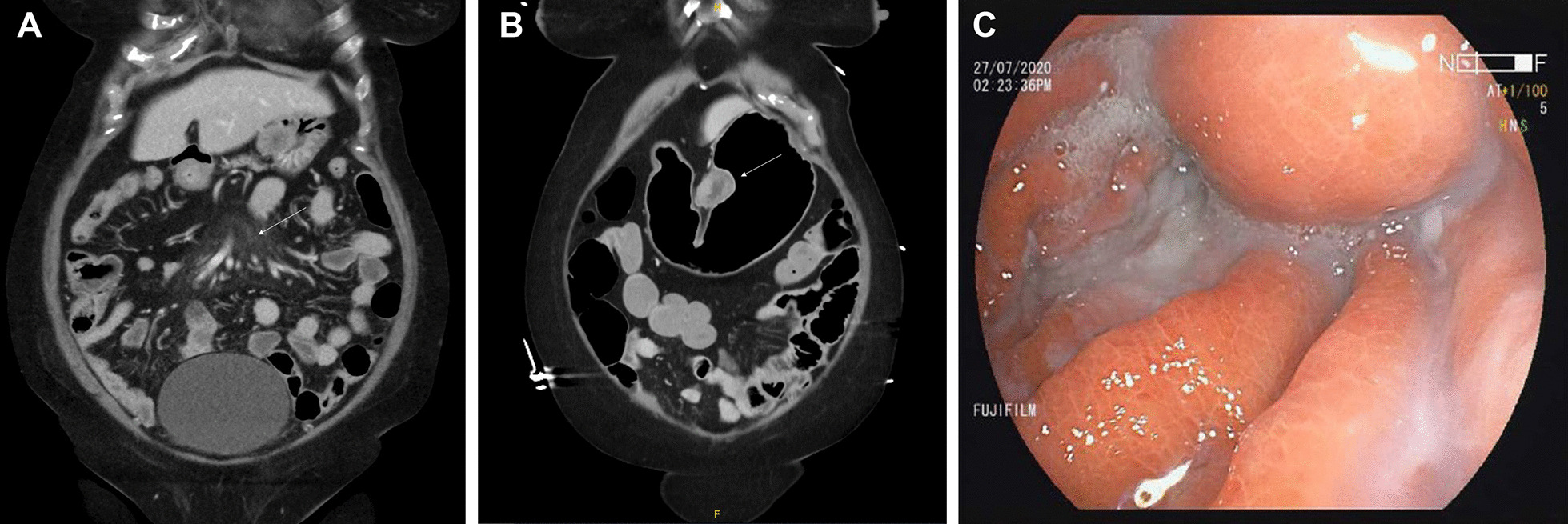
**A** Abdominal computed tomography (CT) on admission revealed misty stranding of the mesentery with small-volume mesenteric nodes suggestive of mesenteric panniculitis. **B** Abdominal CT 20 days later uncovered an intramural mass along the lesser curvature of the stomach. **C** Upper gastrointestinal endoscopy found a gastrointestinal stromal tumor (GIST) along the lesser curve of the stomach

Serum investigations were remarkable for normocytic, normochromic anemia (hemoglobin, 10.6 g/dL), leukocytosis (leukocyte count, 17.1 × 10^9^/L), and an elevated C-reactive protein (CRP, 53 mg/L). In addition, she had mild acute kidney injury (creatinine, 106 umol/L), moderate hyponatremia (sodium, 125 mmol/L), hypophosphatemia (phosphate, 0.78 mmol/L), and hypomagnesemia (magnesium, 0.53 mmol/L). The liver and thyroid function tests were normal. Computed tomography (CT) of the head was unremarkable, with no acute brain infarct or hemorrhage. Conversely, abdominal CT revealed incidental misty stranding of the mesentery with small-volume mesenteric nodes indicative of mesenteric panniculitis (Fig. 1A).

In view of the abdominal CT findings, the general surgeons assessed the patient and suggested empirical treatment for a possible intra-abdominal infection with oral amoxicillin/clavulanate 625 mg three times a day for one week. In addition to antibiotics, the patient was treated with intravenous esomeprazole 40 mg twice a day and ondansetron 4 mg twice a day for three days for her abdominal bloating, and electrolytes were replaced intravenously. Nonetheless, her symptoms did not fully resolve.

**Fig. 2:**
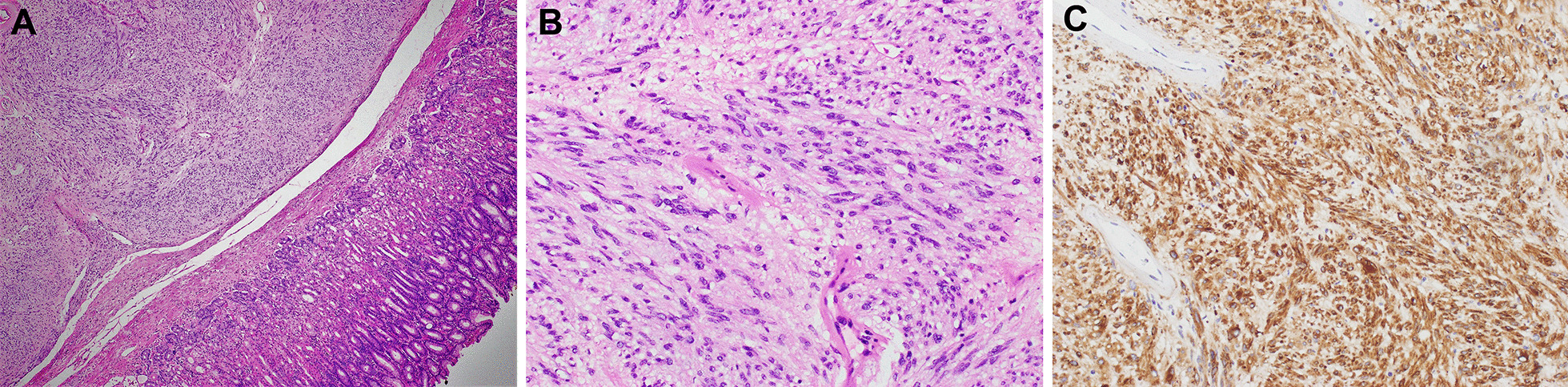
Histopathological images of the resected tumor. **A**, **B** Haematoxylin and eosin-stained tissue showed a low-grade, spindle-type gastrointestinal stromal tumor (GIST) with a histological grade of G1. Original magnifications: **A** ×40 and **B** ×200. **C** The tissue stained positive for tyrosine-protein kinase KIT (also known as cluster of differentiation 117, CD117), confirming the diagnosis of GIST. Original magnification: 200x

Twenty days after her admission, the patient acutely developed small amounts of non-bloody, non-bilious nausea and vomiting after meals, and a repeat abdominal CT revealed an intramural mass along the lesser curvature of the stomach (Fig. 1B). In view of the new symptoms, the general surgeons performed an urgent esophago-gastro-duodenoscopy, which found a 3 cm gastrointestinal stromal tumor (GIST) along the lesser curve of the stomach (Fig. 1C). The patient was transferred to a tertiary hospital for laparoscopic wedge gastrectomy. During surgery, a 3.0 by 2.4 cm gastric GIST with no obvious metastatic deposits was observed. Histological analysis of the resected tissue revealed a low-grade, spindle-cell type GIST with resection margins free of the tumor (Fig. 1A, B). The pathological grade classification was pT2N0, and the histologic grade was G1 with a mitotic rate of 1/5 mm^2^. Immunohistochemical studies were positive for KIT (CD117) and ANO1/DOG1; focally positive for SMA; and negative for S100 (Fig. 2C). Subsequently, after resection of the tumor, the patient reported that her symptoms resolved, and she was able to consume full feeds on post-operative day 3. After a period of rehabilitation at a community hospital, she was discharged home hemodynamically stable, well and satisfied with her care.

Even though the patient reported no more falls or vertigo after her gastrectomy, she subsequently had multiple hospitalizations for nosocomial infections. Three months after her gastrectomy, the patient was re-admitted to hospital for diarrhea due to *Clostridium difficile* colitis. She was successfully treated with a tapering course of oral vancomycin over seven weeks and discharged home. Yet two weeks later, she was admitted again for hospital-acquired pneumonia requiring admission to the intensive care unit. She discharged home after a one-month stay in the hospital, but was physically deconditioned from her recurrent hospitalizations, and required minimal assistance for her activities of daily living and ambulation at home. The patient was reviewed by a community nurse at home nine months after her initial diagnosis of GIST by gastroscopy and reported that her vertigo never recurred after surgery. However, three days after the home visit, the patient suddenly demised at home before a repeat gastroscopy could be performed to evaluate for recurrence of the gastric tumor.

## Discussion and conclusion

Older patients often have multiple comorbidities, age-related physiological changes, reduced body reserves and underreporting of symptoms that contribute to non-specific and atypical presentations of illnesses [7]. Indeed, 34 to 53% of patients aged 65 years and older who attended Emergency Departments have atypical presentations [8, 9]. The most common atypical presentations are falls (71%), cognitive decline (29%), functional decline (11%) and urinary incontinence (3%) [8]. Moreover, other common atypical presentations include anorexia, fatigue, and reduced mobility [9]. In addition to advanced age, non-specific presentations are also associated with cognitive impairment, multimorbidity, frailty, communication problems and institutionalization at a nursing home [8, 9].

Furthermore, older patients may have variant symptoms of specific diseases that differ from the classical symptoms observed in younger patients. For example, in older patients, acute appendicitis may present with diffuse abdominal pain and urinary urgency, without fever or tachycardia [10]. Likewise, acute myocardial infection may have mild or absent chest pain or dyspnea, and absence of typical electrocardiographic changes [11]. Similarly, hyperthyroidism may present with unexplained atrial fibrillation, without weakness or anorexia [12], while complicated urinary tract infections may present with fatigue with no fever or dysuria [13]. Unsurprisingly, many physicians report feeling less confident assessing older adults compared to younger patients [10].

Atypical presentations are associated with poorer quality of care and more adverse outcomes. Due to delayed and missed diagnoses, atypical presentations result in more frequent hospitalizations, increased healthcare costs and higher short-term mortality [8–10]. For instance, 30-day mortality increases from 9.0 to 23.2% in frail older adults hospitalized for pneumonia who have atypical presentations [14]. Furthermore, compared to older adults with typical presentations, community-dwelling older patients hospitalized for atypical presentations are more likely to be discharged to a nursing home (29% vs. 10%) and have longer lengths of stay (12 days vs. 6 days) [8].

Nonetheless, greater awareness of comprehensive approaches to illness presentations in older adults should lead to more accurate and complete diagnoses, and therefore improved outcomes [15]. A careful approach to atypical presentations of acute illnesses involves risk stratification, a broad differential diagnosis, and the interpretation of acute symptoms and investigations in the context of baseline symptoms and prior investigation results [15]. For instance, the evaluation of falls in younger adults is often focused on serious neurological or cardiac etiologies such as strokes, seizures, acute myocardial infarctions, or cardiac arrhythmias. Yet in older adults who tend to have atypical presentations, falls, especially recurrent falls, warrant a thorough evaluation of possible predisposing and precipitating factors. Therefore, a comprehensive geriatric assessment should be performed to evaluate for underlying medical co-morbidities and cognitive impairments; medication side-effects; postural hypotension; visual, auditory, sensory, and proprioceptive impairments; inappropriate footwear and home environments; and occult infections or malignancies [15]. Our case report describes a diagnostically challenging case of an older adult who presented with recurrent falls precipitated by vertigo. A comprehensive geriatric assessment revealed that her giddiness was due to orthostatic hypotension from dehydration due to poor oral intake. The poor oral intake was in turn predisposed by anorexia and abdominal bloating from mesenteric panniculitis associated with a gastric tumor.

It is crucial to not delay diagnosis of upper gastrointestinal malignancies, since early diagnosis and treatment are key to improved mortality and morbidity [16]. Alarm symptoms—weight loss, gastrointestinal bleeding, dysphagia and vomiting—are generally accepted as indications for endoscopy by various international guidelines [1–4], but the positive predictive value of having any alarm symptom is only 5.9% [5], and 25% of individuals with upper gastrointestinal tumors do not display any alarm symptoms [5]. In fact, the most common presenting symptoms for gastric tumors were not alarm symptoms but non-specific symptoms like fatigue (53%) and anorexia (43%) [17]. Older patients in particular often have mild or non-specific presentations, resulting in upper gastrointestinal malignancies being overlooked [6]. A high suspicion for gastric tumors in older adults is also required because, in addition to other risk factors such as East Asian nationality [18–21], male sex [22], and *Helicobacter pylori* infection [23], advanced age is a major risk factor for gastric tumors [22].

While alarm symptoms have limited diagnostic value for gastric tumors, they have been assessed to have prognostic value [24, 25], and are associated with mesenteric panniculitis [26, 27]. The presence of at least one alarm symptom reduces the five-year survival rate by an average of 29% [20, 24, 25], and mortality is three-times higher in patients with an alarm symptom compared to patients with uncomplicated dyspepsia [20]. Mesenteric panniculitis—a rare, chronic fibro-inflammatory condition affecting the mesentery but sparing the adjacent gut, lymph nodes and blood vessels—can cause anorexia, nausea, vomiting, bloating, diarrhea and constipation [26, 27]. Most common in the sixth and seventh decade of life with a prevalence of 0.16–3.3%, mesenteric panniculitis has been linked to autoimmune diseases, infectious diseases and malignancies [27]. Importantly, since mesenteric panniculitis confers a five-fold greater risk of malignancy [28]—such as colorectal, gastric, renal, gynecological, prostate, and hematological cancer—clinicians are advised to search for occult malignancies in symptomatic older adults. Therefore, in addition to age-appropriate cancer screening, gastroscopy should be employed in the hunt for occult gastrointestinal tumors in patients with symptomatic mesenteric panniculitis.

In addition to diagnosis of gastric tumors, gastroscopy can also be employed for the resection of early gastric tumors. While endoscopic resection methods have been associated with higher rates of tumor recurrence, metachronous tumors and incomplete resection compared to gastrectomy, both endoscopic resection and gastrectomy result in similar mortalities and survival rates at three and five years [29]. Moreover, compared to gastrectomy, endoscopic resection for early gastric tumors is associated with lower complication rates, fewer nosocomial infections, and shorter lengths of hospitalization [30]. In retrospect, given the repeated hospitalizations and consequent hospital-acquired infections experienced by the patient, endoscopic resection should be considered for the management of the early-stage GIST for this patient.

On reflection, the history, physical examination, and investigations in our case provided important clues that may have prompted an earlier referral for endoscopy. The patient’s age and East Asian nationality places her at a higher risk for gastric tumors. Even though leukocytosis and raised CRP may be raised during an infection, they also point toward a possible occult tumor. Since the patient’s 3 cm GIST had no tumor rupture or bleeding, it was unlikely to directly cause the patient’s falls and vertigo. On the other hand, the mesenteric panniculitis associated with the GIST likely contributed to abdominal bloating and anorexia. Indeed, the patient’s hypovolemia, hypomagnesemia, hypophosphatemia, and acute kidney injury alludes to anorexia and poor oral intake resulting in electrolyte deficiencies and dehydration. Both electrolyte abnormalities and orthostatic hypotension due to dehydration would have contributed to the patient’s vertigo and falls.

To our knowledge, this is the first reported case of a cryptogenic gastric tumor found by endoscopy in a patient with mesenteric panniculitis, and also the first reported case of an occult gastric tumor associated with mesenteric panniculitis that presented with recurrent falls precipitated by vertigo. This case highlights that older patients tend to present with non-specific or atypical symptoms, such as recurrent falls and giddiness. Atypical presentations make accurate and early diagnosis challenging, and therefore result in adverse outcomes in terms of increased morbidity, mortality, and healthcare costs for patients. Therefore, when older patients develop accelerated geriatric syndromes such as recurrent falls, functional decline or delirium, a careful approach is indicated, including an extended differential diagnosis, a high index of suspicion for occult disease, and risk stratification based on baseline symptoms. A comprehensive approach to illness presentations is especially important in older adults with cognitive impairment, multi-morbidity, frailty, and problems with communication, who are inclined to present with non-specific symptoms.

## Data Availability

Not applicable.

## Abbreviations

ANO1/DOG1: Anoctamin 1
CD117: Cluster of differentiation 117
CRP: C-reactive protein
CT: Computed tomography
GIST: Gastrointestinal stromal tumor
HbA1c: Glycosylated hemoglobin
KIT: Tyrosine-protein kinase
SMA: Smooth muscle actin
